# Transmission or Within-Host Dynamics Driving Pulses of Zoonotic Viruses in Reservoir–Host Populations

**DOI:** 10.1371/journal.pntd.0004796

**Published:** 2016-08-04

**Authors:** Raina K. Plowright, Alison J. Peel, Daniel G. Streicker, Amy T. Gilbert, Hamish McCallum, James Wood, Michelle L. Baker, Olivier Restif

**Affiliations:** 1 Montana State University, Department of Microbiology and Immunology, Bozeman, Montana, United States of America; 2 Center for Infectious Disease Dynamics, Pennsylvania State University, State College, Pennsylvania, United States of America; 3 Environmental Futures Research Institute, Griffith University, Brisbane, Queensland, Australia; 4 Institute of Biodiversity, Animal Health and Comparative Medicine, University of Glasgow, Glasgow, United Kingdom; 5 MRC-University of Glasgow Centre for Virus Research, Glasgow, United Kingdom; 6 USDA/APHIS/WS National Wildlife Research Center, Fort Collins, Colorado, United States of America; 7 Griffith School of Environment, Griffith University, Brisbane, Queensland, Australia; 8 Disease Dynamics Unit, Department of Veterinary Medicine, University of Cambridge, Cambridge, United Kingdom; 9 CSIRO Health and Biosecurity Business Unit, Australian Animal Health Laboratory, Geelong, Victoria, Australia; University of California Berkeley, UNITED STATES

## Abstract

Progress in combatting zoonoses that emerge from wildlife is often constrained by limited knowledge of the biology of pathogens within reservoir hosts. We focus on the host–pathogen dynamics of four emerging viruses associated with bats: Hendra, Nipah, Ebola, and Marburg viruses. Spillover of bat infections to humans and domestic animals often coincides with pulses of viral excretion within bat populations, but the mechanisms driving such pulses are unclear. Three hypotheses dominate current research on these emerging bat infections. First, pulses of viral excretion could reflect seasonal epidemic cycles driven by natural variations in population densities and contact rates among hosts. If lifelong immunity follows recovery, viruses may disappear locally but persist globally through migration; in either case, new outbreaks occur once births replenish the susceptible pool. Second, epidemic cycles could be the result of waning immunity within bats, allowing local circulation of viruses through oscillating herd immunity. Third, pulses could be generated by episodic shedding from persistently infected bats through a combination of physiological and ecological factors. The three scenarios can yield similar patterns in epidemiological surveys, but strategies to predict or manage spillover risk resulting from each scenario will be different. We outline an agenda for research on viruses emerging from bats that would allow for differentiation among the scenarios and inform development of evidence-based interventions to limit threats to human and animal health. These concepts and methods are applicable to a wide range of pathogens that affect humans, domestic animals, and wildlife.

## Pulses of Zoonotic Spillover

Long identified as potential sources of zoonotic pathogens [1,2], bats (Order Chiroptera) are now associated with several deadly emerging infectious viruses, including Hendra, Nipah, Marburg, Ebola, and Severe Acute Respiratory Syndrome coronavirus (SARS CoV). Although spillover from bats to humans or domestic animals remains rare, the case fatality rate from these diseases is high, and onward transmission can occur. Public health preparedness would benefit from understanding bat virus dynamics to allow predictions of viral spillover in space and time.

Spillover of bat viruses is often associated with discrete temporal and spatial pulses of virus excretion from the bats that function as reservoir hosts [3–6]. Outbreaks in livestock or humans occur seasonally with high annual variability. For example, spillover of Hendra virus in Australia, Nipah virus in Bangladesh, Marburg virus in Uganda, and Ebola virus in Central Africa is seasonal, but incidence and location of spillover infections vary among years [5–8]. Longitudinal surveys of bat colonies also have detected seasonal variation in the prevalence or seroprevalence of zoonotic viruses, including Nipah virus in *Pteropus lylei* in Thailand, Hendra virus in *Pteropus* sp. in Australia, and Marburg virus in *Rousettus aegyptiacus* in Uganda [6,9–12].

**Fig 1:**
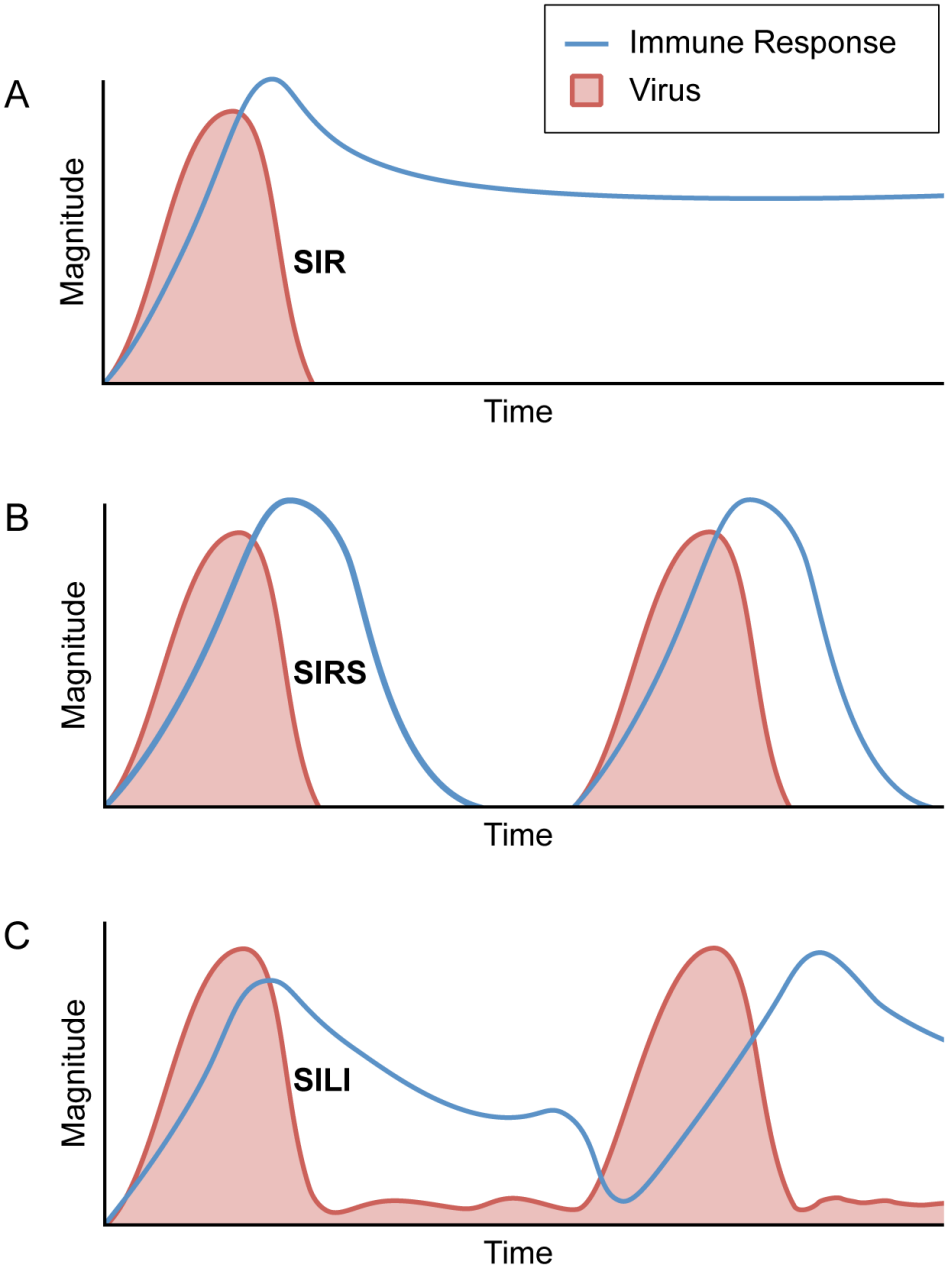
Within-host dynamics. Three working hypotheses represent the range of expert opinion about the dynamics of emerging viruses within bats. (A) Following an initial acute infection, the virus clears completely and bats remain refractory to infection (susceptible-infectious-recovered [SIR]). (B) The virus clears completely, but the bats’ immune response wanes over time, allowing individuals to be reinfected (susceptible-infectious-recovered-susceptible [SIRS]). (C) Following the acute phase of infection, a chronic infection remains, or the infection is latent and then reactivated (susceptible-infectious-latent-infectious [SILI]).

**Fig 2:**
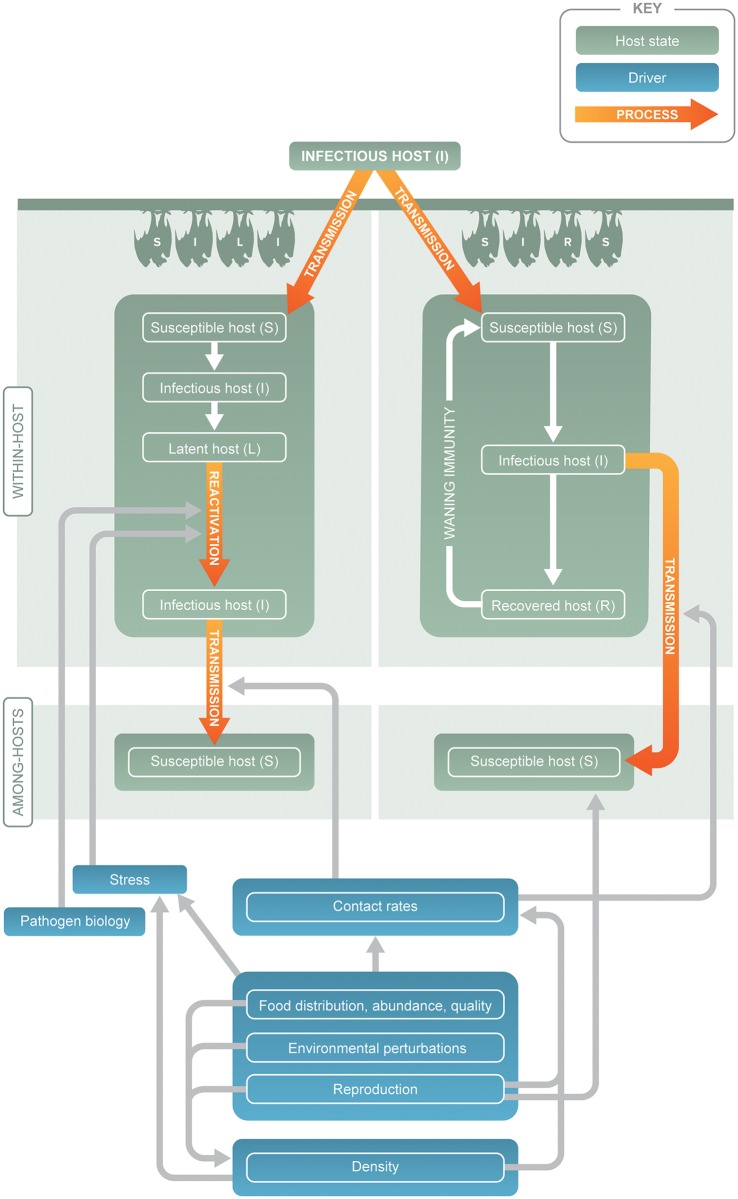
Drivers of disease dynamics within hosts, and within populations, given persistent infections with latency and reactivation (SILI dynamics) or immunizing infections with or without waning immunity (SIR or SIRS dynamics). A common factor among scenarios is seasonal forcing, which occurs through birth pulses, seasonal transmission, or periods of environmental or physiological stress. These factors affect SILI dynamics through reactivation and SIR or SIRS dynamics through transmission.

Markedly different underlying mechanisms can yield similar spatial and temporal patterns in prevalence and seroprevalence data. We outline three distinct scenarios that could generate pulses of viral excretion in bats (Figs 1 and 2). Although the scenarios fall along a continuum, each one leads to a different set of hypotheses that can be tested in the field or laboratory. Within- and between-host processes drive the first and second scenarios: pulses of transmission among bats with clearance of infection and either long-term (Fig 1A) or waning immunity (Fig 1B). Within-host processes drive the third scenario, with pulses triggered by viral reactivation in persistently infected bats (Fig 1C). A common driver among the three scenarios is seasonal forcing, which occurs through birth pulses, seasonal transmission, waning maternal immunity in young, and periods of environmental or physiological stress (Fig 2). Research efforts often reflect the working hypothesis that pulses are driven by between-host transmission or that pulses are driven by within-host processes of reactivation, but often without using data collection methods that would allow them to be distinguished [5,13–16]. In reality, the evidence for either hypothesis has not been fully assessed for the emerging viruses discussed here.

Even though the different scenarios can lead to similar observed dynamics, strategies to predict or manage spillover risk will be different for the different underlying mechanisms (and mechanisms would be expected to vary between virus types). Strategies that confront dynamics driven by transmission between hosts are likely to focus on population density, connectivity among populations, or herd immunity [17]. By contrast, strategies to address dynamics driven by within-host reactivation are likely to target pathogen biology or processes that produce stress [18].

As part of the international focus on surveillance of zoonoses and bat-borne viruses such as Ebola virus, understanding the dynamics of pathogens within animal reservoir hosts should be a research priority [19,20]. However, research on wildlife diseases often focuses on cross-sectional surveillance methods within a limited geographic area and does not capture information that would allow inference to spatial and temporal dynamics. Many studies do not examine the drivers of disease dynamics and therefore cannot draw predictive inferences about the occurrence of zoonotic spillover. Identifying the drivers of bats’ excretion of virus would allow prediction of locations and times when the likelihood of spillover is high, potentially leading to better management or even prevention of spillover.

Here, we establish a framework to assess the evidence for the different mechanisms that could drive the observed epidemiological patterns of emerging bat viruses. We highlight the strengths and limitations of potential investigation methods and recommend ways to distinguish between scenarios, which require ambitious, interdisciplinary research (Table 1). We explain some distinct challenges associated with pathogen studies in bats. Although rabies is an important zoonosis of bat origin, we restrict discussion to the less well-understood emerging bat pathogens. Our concepts are transferable to numerous diseases that affect wildlife, domestic animals, and humans.

**Table 1:** Criteria to differentiate Susceptible-Infectious-Recovered (SIR) dynamics, Susceptible-Infectious-Recovered-Susceptible (SIRS) dynamics, and Susceptible-Infectious-Latent- Infectious (SILI) dynamics in bats; strategies to predict the likelihood of spillover or to minimize the likelihood of spillover for viruses with each type of dynamics; and research that would improve our understanding of bat virus dynamics given each scenario^†^.

Criterion	SIR	SIRS	SILI
Individuals have repeated pulses of excretion.	No	Yes	Yes
Virus genotype is the same in repeated pulses of excretion in individuals.	N/A	No*	Yes
Virus genotypes in multiple pulses of excretion in a population have shared ancestry.	No	Yes	Yes
Virus genotype is different in each population pulse.	Yes*	No prediction	No
Age-specific seroprevalence increases monotonically.**	Yes	Yes**	No**
Waves of infection travel among populations.	Yes	No prediction	No***
Past infection increases the likelihood of present infection. Prevalence of infection among previously positive individuals is higher than among the population.****	N/A	No	Yes
**Information needed for prediction of pulses**	Herd immunity within and among populations	Drivers of contact rates, especially environmental drivers	Drivers of stress, especially environmental drivers
**Intervention strategies**			
Disperse bats	Movement of infectious or susceptible bats could spark epidemics elsewhere; local risk may be neutral or may decrease.		Stress of intervention may increase viral reactivation and shedding.
Cull bats	No effect on risk of spillover if transmission is driven by local density of bats; decreased local risk if transmission is driven by population size.		Stress of intervention may increase viral reactivation and shedding.
**Research agenda**	Monitor herd immunity and metapopulation structure.	Estimate rate of waning immunity; identify contact structure and factors that change contact rates.	Identify drivers of viral reactivation, especially environmental drivers of stress.

^†^SIR, SIRS, and SILI dynamics may be poles on a continuum depending on the time spent in each host state (e.g., an SIRS disease with a long R duration may generate similar dynamics to an SIR disease) and the percentage of individuals that exhibit each dynamic (e.g., if most individuals recover but a few experience SILI dynamics).

*provided there is sufficient resolution in the genotyping.

**assuming antibodies are protective and that studies address multiple epidemics; seroprevalence increases monotonically with SIRS dynamics given particular parameter values.

***waves of invasion may occur for SILI if introduced into naïve connected populations.

**** assuming homogenous transmission dynamics.

## Current Evidence for Potential Mechanisms

Three hurdles have constrained understanding of within-host dynamics of emerging viruses in bats. First, although serological assays often indicate high levels of exposure in bat populations, few studies have isolated the corresponding live virus. For example, Ebola virus [21] and African henipaviruses [22,23] have not yet been isolated from bats despite serological evidence and molecular detection across the range of many African species. Additionally, a SARS-like coronavirus was only recently isolated from bats, nearly a decade after the SARS pandemic [24]. Second, experimental infections may be unsuccessful at reproducing natural infections in bats. For example, after Hendra virus and Nipah virus were isolated, amplified, and experimentally inoculated into bats, viral excretion was rarely detected [25,26], although interpretations were hampered because lifetime infection histories of wild caught bats were unknown. Moreover, most captive infection experiments did not use inoculum derived from wild bats (Table 2, S1 Table), and little is known about doses and routes of infection that mimic exposures in the wild [27]. Third, the necessity of working in biosecurity level 4 conditions constrains the scope and duration of experiments on emerging bat viruses [e.g., 25,28]. For these reasons, many mechanisms that drive infectious disease dynamics in bats, such as viral clearance or persistence, immune memory, and transmission, remain poorly characterized.

**Table 2:** Summary of results from experimental inoculation of bats with emerging bat viruses (see S1 Table for more details and references).

Author	Year	Virus & host species	Inoculum Source	# of Bats	Housed	Transmission Study	Duration of Study	Route of inoculation	Time to excretion (isolate from excreta)	Days viremic (RNA detection from blood)	Days RNA recovered from swabs or excreta	Serological response	Waning immunity w/in study timeframe	Proportion of bats shedding infectious virus	Clinical signs
Williamson et al.	1998	Hendra Virus *Pteropus poliocephalus*	Source n.r., Vero cell passage 5, 10^6.7^ median tissue culture infective dose (TCID_50_)/0.05 mL	11	individually & pairs	Y (horizontal); **not observed**	21–22 days	ON, SQ	n.a.	n.a.	n.a.	**ON infection**: 75% seroconverted by virus neutralization test (VNT) and 100% by enzyme-linked immunosorbent assay (ELISA) on day 21 **SQ infection**: 50% seroconverted by VNT and 100% by ELISA on day 21 **in-contact animals**: no seroconversion by VNT nor ELISA on day 21	N	**0**	None
Williamson et al.	2000	Hendra Virus *P*. *poliocephalus*	Source n.r., Vero cell passage 5, 10^6.7^ TCID_50_/0.05 mL	4	pairs	Y (vertical); **observed 25%**	10–21 days	SQ	n.a. (isolates from organs only)	n.a. (but isolate from buffy coat on day 10)	n.a.	100% seroconverted by VNT and ELISA (days 10–21)	N	**0**	None
Halpin et al.	2011	Hendra Virus *P*. *alecto*	*P*. *poliocephalus*, Vero cell passage 6, 1.5 x 10^7^ TCID_50_/mL	20	individually	N	20–22 days	ON	**6 days (urine isolate)**	**days 5–18**	**throat swab: days 2–7; rectal swab: days 3–15; urine: days 3–19;**	50% seroconverted by VNT (days 10–22)	EQUIVOCAL: 10 bats seroconverted—60% declined by terminal sample; 30% increased by terminal sample; 10% stable at terminal sample	**0.05**	None
Middleton et al.	2007	Nipah Virus *P*. *poliocephalus*	Human patient, Vero cell passage 3	17	individually & pairs	N	3–23 days*	SQ	**12 days (urine isolate)**	n.a. (no blood isolate)	n.d.	100% seroconverted by VNT on day 14/15 (n = 11)	EQUIVOCAL, but suggested by authors	**0.06**	None
Halpin et al.	2011	Nipah Virus *P*. *vampyrus*	Unidentified human patient(s), Vero cell low passage, 5 x 10^5^ TCID_50_/mL	8	individually	N	49–51 days	ON	n.a.	**none**	**throat swab: day 4; rectal swab: day 8**	**initial infection**: 50% seroconverted by VNT (days 14–28); **reinfection**: 88% seropositive by VNT (days 35–51), but authors do not suggest anamnestic response observed	N	**0**	None
Swanepoel et al.	1996	Ebola Virus *Tadarida condylura*, *Tadarida pumila*, *Epomophorus wahlbergi*	Human patient, Vero cell passage 4	>30 (?)	n.r.	N	28 days	SQ	**unclear (isolate from feces on day 21)**	n.a. (but isolates from blood obtained)	n.a.	25% seroconverted by ELISA on day 28	n.d.	n.d. (samples tested in pools)	None
Paweska et al.	2012	Marburg Virus *Rousettus aegyptiacus*	Human patient, Vero cell passage 38, 10^4^ TCID_50_/mL	30	groups, 2–4	N	21–29 days	ON, SQ/IP	n.a. (isolates from organs only)	**days 5–9 (SQ/IP only)**	**none**	**initial infection (SQ/IP group only)**: >75% seroconverted by ELISA (see Fig 5, precise proportion n.r.); **initial infection (SQ/IP group only)**: 33% seroconverted by VNT on day 21; **reinfection**: antibody response n.r.	EQUIVOCAL	**0**	None
Paweska et al.	2015	Marburg Virus *R*. *aegyptiacus*	Human patient, Vero cell passage 2, 10^5.3^ TCID_50_/mL	36	groups, 6	Y (horizontal); **not observed**	42–58 days	SQ	n.a. (isolates from organs only)	**days 3–12 (infected bats only)**	**oral swab: days 7–10; vaginal swabs: days 4–21; rectal swabs: days 3–4; urine: days 7–9**	**initial infection**: 100% seroconverted by ELISA on day 14 (n = 22); **in-contact animals**: no seroconversion (n = 14); **reinfection** (n = 4): anamnestic response observed	Y	**0**	None
Amman et al.	2015	Marburg Virus *R*. *aegyptiacus*	*R*. *aegyptiacus*, Vero cell passage 2	30	groups, ≤9	N	3–28 days*	SQ	**8 days (oral swab isolates, 2 bats)**	**days 1–9 (infected bats only)**	**oral swab: days 4–14; rectal swabs: days 4–11;**	**infected animals**: 100% seroconverted by ELISA on day 12 (n = 6);	N	**0.11**	None
Jones et al.	2015	Marburg Virus *R*. *aegyptiacus*	*R*. *aegyptiacus*, Vero cell passage 2	5	groups, 2–4	N	5–10 days*	SQ	n.a.	**days 4–8 (infected bats only)**	**oral swabs: days 7–10; rectal swabs: days 6–10;**	n.a.	n.a.	n.a.	None
Jones et al.	2015	Marburg Virus *R*. *aegyptiacus*	*R*. *aegyptiacus*, Vero cell passage 2	6	groups, 3–9	N	15 days	SQ	n.a.	**days 5–8 (infected bats only)**	n.d.	n.d.	n.a.	n.a.	None
Jones et al.	2015	Ebola Virus (five strains) *R*. *aegyptiacus*	**Ebola Virus**: Human patient(s), Vero cell passage 2; **Sudan Virus**: Human patient(s), Vero cell passage 3; **Bundibugyo Virus**: Human patient(s), Vero cell passage 2; **Tai Forest Virus**: Human patient(s), Vero cell passage 5, **Reston Virus**: Rhesus macaque, MA104 passage 1, Vero cell passage 7	21	groups, 2–4	N	5–10 days*	SQ	n.a.	**none**	**none**	n.a.	n.a.	n.a.	None
Jones et al.	2015	Ebola Virus *R*. *aegyptiacus*	Sudan Virus: Human patient(s), Vero cell passage 3	15	groups, 3–9	N	3–15 days*	SQ	n.a.	**none**	**none**	17% seroconverted by ELISA on day 12; 66% on day 15	n.d.	n.a.	None
Paweska et al.	2016	Ebola Virus R. aegyptiacus	Human patient, Vero cell passage 4	24	groups, 6	Y (horizontal); **not observed**	3–37*	SQ	n.a.	**day 3**	**none**	33% seroconverted by ELISA on day 10; 100% on day 14	Y, marginal	**0**	None
Paweska et al.	2016	Ebola Virus R. aegyptiacus	Human patient, Vero cell passage 4	11	n.r.	N	5–16*	IP,IM	n.a.	**day 7 (IP), days 7–16 (IM)**	**none**	80% seroconverted by ELISA on day 16	n.d.	**0**	None

n.d. = not determined

n.a. = not applicable

n.r. = not reported

* = euthanasia at serial time points

SQ = subcutaneous, IP = intraperitoneal, IM = intramuscular, ON = oronasal

### Virus clearance from hosts

Experimental infections of Hendra, Nipah, and Marburg viruses in bats have suggested that, after a short incubation period, bats have an acute systemic phase of viral replication (Table 2, S1 Table). RNA from Hendra virus was detected in urine and feces for an average of seven days [25]; Hendra virus was isolated from bat tissues 10 days, but not 21 days, after infection [29,30]. Likewise, in three Marburg virus experiments, bats experienced a short period of viremia followed by clearance from most sampled tissues by 10 to 14 days after inoculation [31–33]. There were no signs of morbidity or mortality induced by Hendra, Nipah, Ebola, or Marburg viruses, consistent with previous virus detections in apparently healthy wild bats (Table 2) [6,33–35]. However, all henipavirus experiments and one Marburg virus experiment were conducted with individual wild-caught bats without knowledge of the prior infection and immune histories.

The sequelae to the acute period of viral infection and shedding in bats are less well characterized. Although virus was not isolated from bats at the conclusion of any experiment, RNA was frequently detected in tissues at necropsy (S1 Table). For instance, Hendra virus RNA was detected in the lung, liver, spleen, and kidney of bats at the conclusion of a three-week experiment [25]. Marburg virus RNA was detected in the spleen at the conclusion of one four-week experiment [31] but was cleared from all sampled tissues at the conclusion of another three-week experiment [32]. To understand viral clearance, two important questions must be resolved. First, is the absence of PCR detection sufficient to demonstrate clearance? Measles virus RNA, for example, can be detected in humans for months after active infection has cleared [36]. Second, what experimental duration is adequate for assessing viral persistence?

### Virus persistence within hosts

Although there is no direct evidence that the henipaviruses or filoviruses persist within individual bats, it is commonly assumed that bats host persistent infections [37]. This assumption may be derived from evidence of persistent flavivirus and Rio Bravo virus infections during experiments conducted in the 1960s and 1970s [38,39], and a common paradigm that reservoir hosts carry persistent infections (e.g., hantavirus in rodents and simian immunodeficiency virus [SIV] in primates [40,41]). More recently, simultaneous shedding of several viruses (and sometimes bacteria) from many individuals was observed during pulses of excretion [e.g., 42–44]; this observation could be explained by within-host persistence. Some features of the immune systems of bats appear to be different from those of other mammals, and this has fueled speculation that bats may host persistent infections [16]. For example, the set point of interferon responses in bats appears to be relatively high compared to other mammals [45]. The latter is consistent with the possibility that bats rapidly control viral replication, thus avoiding the pathological consequences of disease observed in other species [45]. Moreover, it has been proposed that bats can coexist with pathogens due to adaptations inadvertently acquired during the evolution of flight [46–48]. Despite this growing body of research on bat–pathogen interactions, there is no direct evidence that henipaviruses or filoviruses persistently infect their bat hosts.

If bats do not clear infections, there are at least two mechanisms by which viruses may persist within bat hosts [18]. First, bat hosts may tolerate viruses. In this case, a tempered inflammatory response would minimize immunopathology; viral replication may fluctuate as a function of the immunological competence of the host [18]. Second, persistent latent or low-level infections from which virus can be reactivated may become established in bats [13]. In theory, persistent bat viruses could reactivate in response to processes that affect host immunity, such as stress, pregnancy, or poor nutrition [5,14]. Whether a particular individual clears infection or remains persistently or latently infected may depend on immune status, dose received, coinfections, route of infection, history of infection, and individual variation—all factors that may differ amongst individuals and through time [18].

Latent and recrudescent Hendra and Nipah encephalitis cases were observed in humans, and, therefore, recrudescence was proposed as a mechanism of persistence in bats. However, recrudescent virus in humans was not transmitted and, therefore, did not contribute to epidemiological dynamics [e.g., 49,50–52]. Nevertheless, the different cellular interactions between viruses and reservoir hosts versus incidental hosts may lead to different mechanisms of persistence. For example, the ability of bats to control viral replication may limit the capacity of viruses such as Hendra and Nipah to spread to the central nervous system of bats. In general, characterization of host pathogen dynamics in a spillover host does not provide evidence for the dynamics being similar in reservoir hosts [5,27]. Recrudescent Nipah virus infection was reported in a bat, but the evidence presented was weak and alternative explanations were not ruled out [13].

### Protective immunity

Experimental evidence for protective immunity following viral infection in bats is inconsistent and suggests differences among species and viruses (Table 2). In the 1960s Sulkin et al. [53] described recurrent viremias in bats and susceptibility to reinfection with Japanese B encephalitis virus, establishing a paradigm for bats as hosts of recurrent infections. By contrast, repeat challenge studies with Rabies virus in one bat species confronted our assumptions about the universal lethality of rabies virus in mammals. Although some individual bats experienced acute, lethal infections, other individuals survived with protective immunity and never shed virus [54]. Contemporary work on emerging bat viruses has been less conclusive. Bats challenged with Marburg virus seroconverted and were protected from viral replication when rechallenged 48 days later, suggesting some short-term protection after recovery from acute viremia [32]. In contrast, 50% of bats inoculated with Hendra or Nipah virus seroconverted, and there was no consistent relation between seroconversion and recovery of viral genome [25]. Moreover, in another study, bats excreted Nipah virus in urine while neutralizing antibody was present in the serum [26]. Together, these studies indicated that antibodies may not be the primary driver of henipavirus clearance in bats [55]. Reinfection experiments with Nipah virus were inconclusive; most bats did not respond to the first inoculation with productive infections [25]. Waning prevalence of Hendra virus antibody was reported in a maternity colony of *Pteropus scapulatus* over six months [9], and waning maternal henipavirus antibodies have been reported in numerous systems [e.g., 9,56,57], but the effective protection conferred by these antibodies was not determined. The association between antibodies and Ebola viral nucleic acids in samples from bats was unclear [58].

### Transmission

As is the case with most wildlife diseases, the process by which bat viruses are transmitted in the wild is poorly understood. Transmission events cannot be observed directly, viral infections of reservoir hosts may be asymptomatic, and sampling of individuals through time is difficult. Therefore, inferences must be drawn from multiple sources of indirect evidence. Bat viruses have been detected in urine, feces, saliva, and soiled fruit, indicating the potential for virus transmission via excreta between bats in the wild [5,59]. However, under laboratory conditions, bat-to-bat transmission for most of these viruses is difficult to achieve (Table 2) [e.g., 32]. Following experimental inoculations, viral shedding by bats for both Nipah and Hendra viruses was minimal [25], although continuous exposure to a low-dose viral rain may increase the probability of infection [5]. Natural and experimental vertical transmission of Hendra virus has been documented, but inconsistently and under specific conditions, such as parenteral inoculation (but not oronasal inoculation) [25,29]. A few longitudinal serological and virological surveys and theoretical models have proposed that seasonal patterns in incidence or spillover could be explained by peaks of transmission in response to demographic or ecological drivers such as birth pulses, waning maternal immunity, and migration [12,15,60,61], but these proposed dynamics have not been confirmed.

## Three Scenarios for Pulses of Infection

Disease dynamics within populations are primarily driven by interactions among the processes of pathogen transmission and clearance, immunity, and host population dynamics. We focus on three different scenarios—representing different combinations of these within- and between-host processes—that could generate pulses of viral excretion in bat populations (Fig 2). Although we present these scenarios as discrete, they fall along a continuum. Our purpose in proposing these scenarios is to inform research on the relative importance of key components of these systems to allow for generation of testable hypotheses. Simulations combined with field and laboratory studies can then be used to assess the relative explanatory capacity of the three models.

The first scenario is that pulses of infection are driven by transmission of short-lived infections that provide long-lasting immunity (Fig 1A, susceptible-infectious-recovered [SIR] dynamics). Two paramyxoviruses, measles virus and distemper virus, are emblematic SIR diseases in human and animal epidemiology [17]. The dynamics of Hendra virus and Nipah virus, which are also paramyxoviruses, have been assumed to be similar to those of measles and distemper [e.g., 15]. A recent model of filoviruses in bats similarly assumed that SIR dynamics characterized Ebola and Marburg viruses in bats [60]. In the absence of antigenic evolution, SIR pathogens render recovered hosts immune. Therefore, epidemic pulses can only occur after births or immigration have replenished the pool of susceptible individuals [17]. Large, regular outbreaks of zoonotic viruses within bat populations could occur through local viral extinction and then recolonization, seasonal aggregations, or the influx of susceptible juveniles [5,15,60].

In the second scenario, pulses of infection are driven by transmission of short-lived infections with fluctuating host immunity (Fig 1B, susceptible-infectious-recovered-susceptible [SIRS] dynamics). The pool of susceptible individuals is replenished via antibody decay in immunized hosts. In this scenario, although births or immigration contribute to the cycles of infection, loss of immunity within individuals strongly affects the age-specific incidence. Replacement of susceptible individuals from within populations also contributes to endemic persistence of pathogens in small populations. Whether this scenario may occur in bats has not been explored. However, it could explain short intervals between pulses of excretion of viruses, such as Hendra virus [12], that would be unlikely under SIR dynamics. If SIR dynamics explain pulses of infection, then more time between pulses would be necessary to replenish susceptible individuals.

The third scenario hinges on resolution of the acute infection without clearance of virus, allowing pulses of transmission to be triggered by viral reactivation (Fig 1C, we call these dynamics susceptible-infectious-latent-infectious [SILI]). Under this scenario, pathogen carriage is stable in space and time even if viral shedding is episodic [18]. The synchrony of shedding among bats may depend on the extent and synchrony of the drivers of shedding (Fig 2, e.g., food shortages or climate conditions), the abundance of susceptible individuals (e.g., during a birth pulse [61]), and behaviors that increase transmission rates [15].

Each of these scenarios can be considered as discrete examples along a continuum. For example, diseases driven by SIR and SIRS dynamics may have similar patterns if the duration of the R state is long in the SIRS case. Diseases driven by SILI and SIR dynamics may have similar patterns if the duration of the I state is long in the SIR case. Moreover, SIR, SIRS, and SILI dynamics may not be mutually exclusive; they might even co-occur within or among heterogeneous populations.

## Research Methods

Each of the three scenarios outlined above may generate similar patterns in prevalence or seroprevalence data. Research methods that can be combined to distinguish among these scenarios include field sampling to document seroprevalence and prevalence, statistical modeling to identify patterns of infection, pathogen sequencing and phylogenetic analysis, and dynamic simulation modeling to generate epidemiological scenarios that can be evaluated with data.

### Field sampling of seroprevalence

Quantification of seroprevalence often has been the first line of investigation for newly discovered bat zoonoses [e.g., 8,9,11]. Serological assays have some limitations [62], but antibodies are generally easier to detect than antigen; sera can be nonlethally sampled, and, because antibodies generally persist after virus is cleared, the probability of detecting seropositive animals is higher than that of detecting infected animals. However, seroprevalence is a poor metric for quantifying epidemic dynamics. If antibodies persist within bats, seroprevalence measures cumulative exposure. If antibody persistence exceeds the period over which hosts move between populations, then antibody prevalence may not reflect the epidemic history of the sampled population, but simply the infection history of the individual [63]. Thus, longitudinal monitoring of seroprevalence may be most useful for studying the dynamics of viruses that produce short-lived antibody responses in relatively sedentary host species (unlike Pteropid bats).

Measuring age-specific serostatus and antibody titers increases the power of serological surveys. If age-specific seroprevalence increases monotonically as age increases in populations sampled over multiple outbreaks, SIR dynamics can be inferred (Table 1). In contrast, SIRS and SILI dynamics could exhibit complex patterns in cross-sectional serosurveys. These patterns would change through time as epidemics wax and wane. In general, inference from serology alone is unlikely to differentiate among our proposed epidemiological scenarios.

### Field sampling of infection prevalence

The difficulty of estimating prevalence of emerging bat viruses may reflect true low prevalence, low test sensitivity, or sampling of tissues in which virus does not persist. Pathogens that cause acute infections may circulate at low prevalence or may be heterogeneously distributed in space and time and therefore require large sample sizes to detect [64].

Pathogens that persist within their hosts may be sequestered in tissues that are difficult to sample non-lethally. For example, Ebola virus RNA has only been detected in the liver and spleen of wild bats [58]. Viremia or shedding in excreta may be periodic, and, therefore, estimation of infection status from these samples may yield false negatives.

Prevalence data collected across space and time may allow identification of geographic patterns, such as travelling waves, that are likely to be caused by host-to-host transmission (SIR or SIRS dynamics) (Table 1). Age-structured prevalence data with an age-specific incidence skewed towards younger individuals suggests an endemic disease with SIR dynamics. If the duration of maternal-derived immunity is known, the mean age of infection is a direct reflection of the basic reproductive ratio of the disease in the population. Although prevalence data provides many insights into the dynamics of disease, there are few situations in which prevalence data alone can distinguish among our proposed epidemiological scenarios.

### Longitudinal sampling of individuals

Longitudinal sampling of the infection status of individuals can help distinguish between scenarios if four conditions are met: (1) Prevalence of the infection must be high enough to detect pathogen within a small number of sampled individuals but must not approach 100% so that variations can be observed. (2) The period over which individuals are resampled must exceed that of the infectious period (and therefore the infectious period must be known). (3) The host’s lifetime must far exceed the infectious period. (4) Pathogen must be detectable without lethal sampling. It is our contention that, if these conditions are met, it should be possible to distinguish among scenarios. In a population with waning immunity and cyclical reinfection, the probability that a given individual becomes infected is expected to be equal to the prevalence of infection among other individuals of similar age and the same sex. Therefore, past infection status is not expected to predict present infection status. In contrast, if individuals are persistently infected but shed episodically, the prevalence of infection among previously positive individuals will be higher than among the population, and past infection increases the likelihood of positive infection status (Table 1). However, large sample sizes and intermediate levels of prevalence probably are required to distinguish cyclical reinfection from persistent infections, and alternative explanations for patterns of prevalence may be difficult to reject. For example, the individuals most likely to be exposed may appear to be persistently infected. In this case, complementing longitudinal sampling with sequence data may improve inferences.

A caveat to this method is that longitudinal, systematic sampling of wild animals, particularly highly mobile or migratory species, requires major effort. For example, large populations of *Eidolon helvum* (the reservoir hosts of henipaviruses and Lagos bat virus) migrate across continents [22]. Even in resident populations, such as urban populations of *Pteropus alecto* (the reservoir host of Hendra virus), recapture of individuals within populations that include tens of thousands of individuals is difficult if not impossible. In contrast to studies in migratory canopy-dwelling species, capture-mark-recapture methods have been implemented in cave-dwelling species. For example, Frick et al. [65] reported recapture rates of 0.10 to 0.35 during longitudinal studies of *Myotis lucifugusi*, and Smith et al. [66] reported recapture rates of up to 0.81 during longitudinal studies of *Myotis macropus*.

If recapture is possible, generating a consistent time-series of recaptures may be challenging, affecting the ability to make inferences on the temporal resolution of the underlying processes. Nevertheless, some mechanisms may be ruled out even with incomplete time-series data. For example, SIR dynamics are unlikely in an animal in which virus is detected, not detected, and then detected again. However, genomics still is necessary to differentiate SIRS from SILI dynamics.

### Viral sequencing

**Fig 3:**
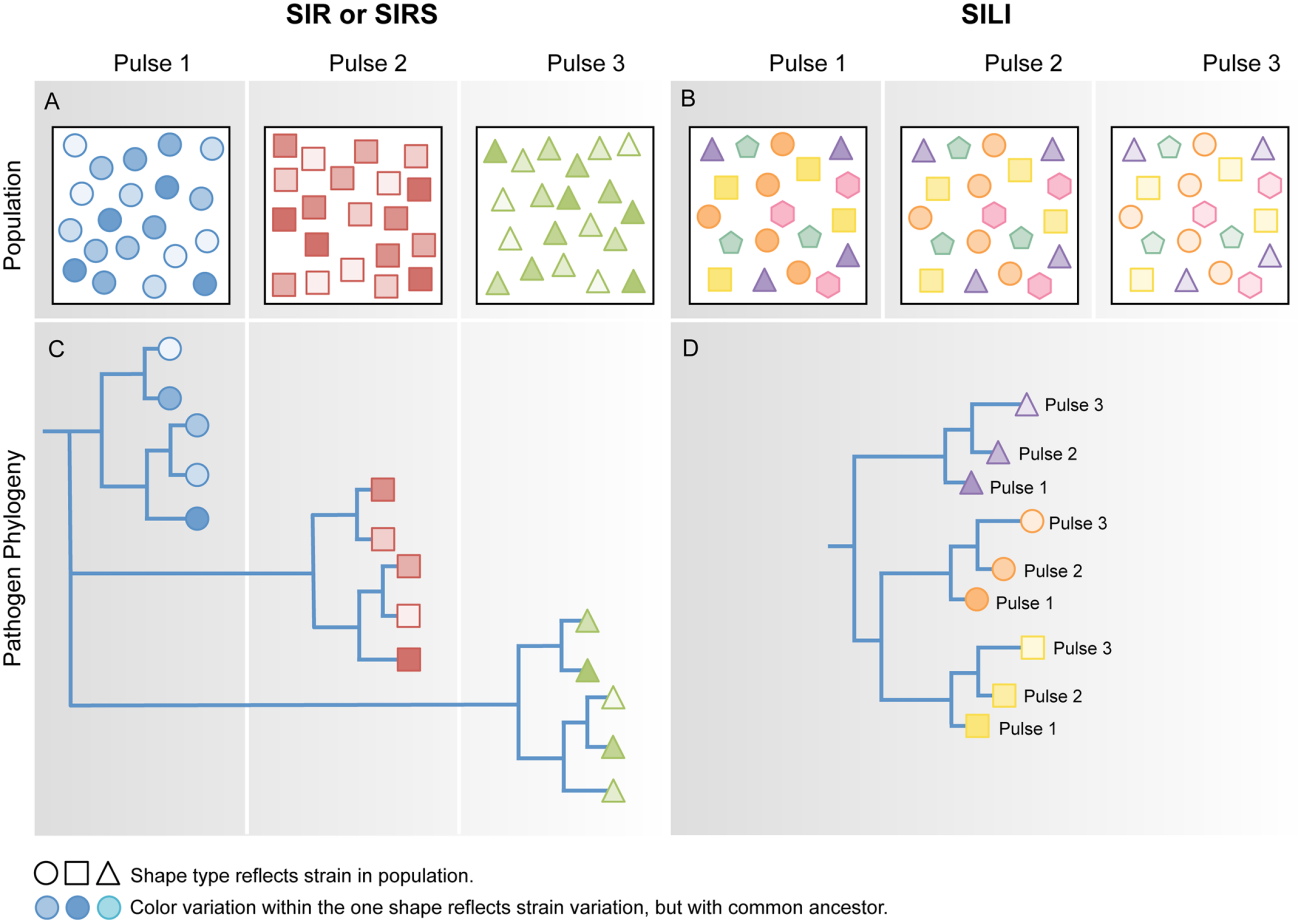
Different within- and between-host mechanisms are hypothesized to produce different evolutionary patterns of viral diversity at the level of populations and individuals. Different shapes represent different viral strains within a population and different colors within a shape reflect variation within strains that have a recent common ancestor. (A) In the case of SIR/SIRS dynamics, acute infections are reintroduced and then cleared at the population level between each pulse. At each point in time, the pathogen within individuals in the population either has the same genotype (e.g., blue circles at pulse 1, red squares at pulse 2) or has closely related genotypes with a common ancestor (matching shape but different color). (B) In the case of SILI dynamics, individuals remain infected over time. Genetic diversity is determined in part by within-host viral evolution. Therefore, genotypes are likely to differ among individuals (many unique shape and color combinations, with some consistency over time). (C, D) Illustrative phylogenies of the virus populations across pulses. (C) In a scenario of viral extinction and reintroduction, all strains at a given pulse are closely related and have a recent common ancestor. (D) Divergent strains (different symbols) may be detectable within pulses, and distinctive strains are maintained across pulses. The hexagon and pentagon represent unsampled viral diversity present in the population.

We hypothesize that pulses of infection arising from reactivation of persistent viruses or reintroduction of acute viruses may leave distinguishable evolutionary signatures in viral genomes at the population and individual levels (Fig 3). Viral reintroduction followed by extinction could result in pulses with low levels of viral diversity (Fig 3A). In contrast, pulses driven by reactivation may contain higher viral diversity given the longer evolutionary history of the virus in the population (Fig 3B). The opposite pattern is expected in comparisons of viral diversity across multiple pulses. Each pulse of reintroduced viruses is more likely to represent a different viral lineage, but reactivated viruses may be similar among pulses. Therefore, reintroduced pulses are expected to create low lineage diversity within an outbreak, but higher lineage diversity among outbreaks. The opposite could be expected for pulses driven by reactivation. For example, diverse lineages of Marburg virus circulated simultaneously within and among bat colonies in Uganda across years [67], a pattern that is more consistent with persistence within hosts and reactivation than pulses of reintroduction, which would require simultaneous extinction-recolonization of multiple strains. Information on viral diversity should ideally be coupled with knowledge of the landscape-level and host community-level distribution of viral lineages, enabling inference to the geographic or species origins of viral reintroductions to focal populations [68].

In the absence of comprehensive, landscape-level data, other features of viral evolution may help distinguish reactivating from recolonizing viruses. Because viral evolutionary rates can be estimated for many species or can be approximated from better-studied related viruses [69], internal branches of the phylogenetic tree connecting pulses of infection in the same bat population may be too long to be plausible under a model of reactivation. Moreover, because neutral evolutionary rates are likely to be slow while viruses are latent, even less evolutionary distance would be expected between pulses driven by reactivation compared to viruses that circulate continuously over the same timespan [70,71]. Predictions of coalescent theory may also help identify recolonizing viruses because viral effective population size is expected to be driven by transmission between individuals [72]. Therefore, effective population sizes are expected to increase exponentially during pulses driven by reintroduction and between-host transmission but may appear stable in pulses driven by reactivation [73]. However, this effect may dissipate if reactivated infections of a few individuals drive a more extensive outbreak with between-individual transmission (i.e., pulses that are driven by multiple mechanisms). Also, as with any such analysis, reconstructions of effective population size depend on a number of epidemiological and evolutionary factors (e.g., selection) as well as sampling effort, so they must be considered with some caution [72].

Repeated sampling of individuals, although challenging and sometimes impossible, may also provide powerful insights. In pulses of reintroduced viruses, the individual viruses sampled at different points in time might be paraphyletic (i.e., recent samples are not necessarily derived from older samples in a phylogenetic tree), whereas reactivated viruses are expected to be monophyletic, with differences among pulses attributable to within-host evolution (Fig 3). Combining deep-sequencing methods that can characterize mixed-strain infections with repeat sampling could further indicate whether lineages are lost and reintroduced or maintained in individuals over time [74].

Viral sequence data may therefore help to distinguish the mechanisms driving pulses of viral infection in bats; however, several aspects of viral evolution present challenges. First, patterns of evolution within and between hosts are just beginning to be understood and different processes can generate similar patterns [73,75]. For example, strong within-host selection and viral reintroduction could create temporal structure in the virus phylogeny [76]. In this regard, sequencing both neutrally evolving and selected parts of the viral genome would be advantageous and is increasingly practical with advances in whole genome sequencing [74]. These methods will be most powerful in rapidly evolving RNA viruses that are temporally and spatially sampled, but this requires major investment in field sampling of highly mobile species like bats, particularly when the prevalence of detection of viral genomes can be low. Even when abundant molecular data are available, experiments and modeling should be designed to complement field sampling and sequencing of RNA viruses over space and time, particularly in more complex epidemiological scenarios in which our hypothesized expectations may not be realized or detectable.

### Numerical exploration of epidemiological scenarios

In the face of incomplete data, modeling is regularly applied to explore the dynamics that would be expected given different scenarios for within- and between-host processes. For example, Blackwood et al. [77] compared the strength of evidence for four epidemiological models of rabies in vampire bats. They concluded that abortive immunizing infections and metapopulation dynamics contribute to population-level maintenance. Assuming an SIR scenario for Filoviruses in *Rousettus aegyptiacus* bats, Hayman [60] investigated the relative importance of parameters controlling bat life history and infection dynamics, highlighting the key role played by the biannual birth pulses that are characteristic of this species. Plowright et al. [15] used models to demonstrate that Hendra virus prevalence and seroprevalence studies were inadequate to distinguish between SIR or SILI dynamics in fruit bats. Although models cannot prove a certain epidemiological scenario exists, they can identify patterns that may help distinguish between scenarios, and therefore parameters, that are important to measure in the natural systems. It is important to reiterate that, to remain useful, models should be revised throughout the course of research programs to include the latest updates from empirical data and any new scenarios deemed relevant [78,79].

## Summary

Effective interventions to limit threats to human health from emerging infectious diseases rely on understanding the connections among observed pathogen dynamics, the underlying transmission mechanisms, and their ecological and environmental drivers. We propose that multiple processes can lead to similar spatial and temporal patterns in prevalence and seroprevalence data, and, to our knowledge, existing empirical data often are insufficient to identify the underlying mechanisms. We encourage researchers to collect appropriate data to identify the scenarios driving pulses of excretion of emerging bat viruses because strategies to predict or reduce spillover risk should depend on the mechanisms’ underlying dynamics (Table 1). Current management strategies include dispersing bats, culling bats, preventing contact between bats and spillover hosts, or vaccinating spillover hosts [80,81]. With the exception of equine vaccination for Hendra virus, few data exist on the efficacy of these strategies. Simulations combined with knowledge of bat virus dynamics can be used to assess the relative effectiveness of each strategy and identify situations in which management action may be counterproductive. For example, culling bats may decrease spillover risk if transmission of the virus increases as population size increases (SIR dynamics), whereas culling may increase spillover risk if pulses are driven by viral reactivation after stress (SILI dynamics). Dispersing bat colonies has clear potential for unintended consequences if pulses are driven by recolonization (SIR dynamics) or by viral reactivation (SILI dynamics). Predicting pulses driven by SIR, SIRS, or SILI dynamics may require information on herd immunity, within- and between-group transmission, and environmental stress, respectively. Thus, the research with the greatest potential to inform prediction and management of spillover varies among scenarios (Table 1).

Identifying the mechanisms and drivers of pulses of virus excretion may be the greatest challenge to predicting, managing, and preventing spillover of emerging diseases. We suggest that genomics and longitudinal sampling of individuals be integrated into virus and antibody surveys, and that these data be analyzed with inferential models. These methods will allow for identification of the drivers of infection pulses and thus set the agenda for research on emerging viruses that originate from bats. Moreover, these techniques can be applied to a range of animal and human systems in which differentiating between- from within-host mechanisms is challenging.

**Key Learning Points**

- A limited knowledge of the biology of pathogens within reservoir hosts makes understanding, predicting, and managing zoonoses that emerge from wildlife very challenging.
- One challenge is that different mechanisms can yield similar patterns in epidemiological data, yet strategies to predict or manage spillover risk will be different for each. Therefore, distinguishing among mechanisms driving disease dynamics in reservoir hosts may be critical for developing public health interventions.
- Spillover of emerging bat viruses to humans often coincides with pulses of viral excretion from bat populations. We present three scenarios of within-host and between-host processes that can generate these pulses of virus excretion. Each scenario involves different underlying mechanisms, yet can produce similar patterns in prevalence data. Research efforts often do not use data collection methods that would allow these mechanisms to be distinguished.
- We show that understanding the connections among observed pathogen dynamics, the underlying mechanisms, and their ecological and environmental drivers requires ambitious, interdisciplinary research. We suggest a research agenda combining genomics, longitudinal sampling of individuals, virus and antibody surveys, and inferential models.

**Top Five Papers**

1. Zhou P, Tachedjian M, Wynne JW, Boyd V, Cui J, Smith I, et al. Contraction of the type I IFN locus and unusual constitutive expression of IFN-α in bats. Proceedings of the National Academy of Sciences. 2016;113(10):2696–701. 10.1073/pnas.1518240113.
2. Plowright RK, P. Eby, P. J. Hudson, I. L. Smith, D. Westcott, W. L. Bryden, et al. Ecological dynamics of emerging bat virus spillover. Proceedings of the Royal Society of London B: Biological Sciences. 2015;282(1798):20142124.
3. Amman BR, Carroll SA, Reed ZD, Sealy TK, Balinandi S, Swanepoel R, et al. Seasonal pulses of Marburg virus circulation in juvenile Rousettus aegyptiacus bats coincide with periods of increased risk of human infection. *PLoS Pathog*. 2012;8(10):e1002877.
4. Luby SP, Hossain MJ, Gurley ES, Ahmed B-N, Banu S, Khan SU, et al. Recurrent zoonotic transmission of Nipah virus into humans, Bangladesh, 2001–2007. Emerging infectious diseases. 2009;15(8):1229.
5. Restif O, Hayman DTS, Pulliam JRC, Plowright RK, George DB, Luis AD, et al. Model-guided fieldwork: practical guidelines for multidisciplinary research on wildlife ecological and epidemiological dynamics. Ecology Letters. 2012;15(10):1083–94.

## Supporting Information

S1 Table Detailed summary of results from experimental innoculation of bats with emerging bat viruses. (XLSX)
